# The validation of the Norwegian Basic Psychological Need Satisfaction and Frustration Scale: A stratified sampling procedure

**DOI:** 10.3389/fpsyg.2022.1032006

**Published:** 2022-10-13

**Authors:** Jolene van der Kaap-Deeder, Alba Sanchez, Maria Regine Aasland Johannessen, Frode Stenseng, Ingvild Saksvik-Lehouillier, Andreas Heissel

**Affiliations:** ^1^Department of Psychology, Norwegian University of Science and Technology, Trondheim, Norway; ^2^Social and Preventive Medicine, Department of Sports and Health Sciences, Faculty of Human Science, University of Potsdam, Potsdam, Germany; ^3^Social- and Preventive Medicine, Intra-Faculty Unit, Cognitive Sciences, Department of Sports and Health Sciences, Faculty of Human Science, and Faculty of Health Sciences Brandenburg, Research Area Services Research and e-Health, University of Potsdam, Potsdam, Germany

**Keywords:** basic psychological needs, self-determination theory, mental health, validation study, basic psychological need satisfaction and frustration scale

## Abstract

This study aimed to validate the Norwegian version of the Basic Psychological Need Satisfaction and Frustration Scale (BPNSFS) and to examine its relations with indicators of well-being and ill-being. Additionally, despite the vast number of studies employing the BPNSFS, norms related to the BPNSFS are currently lacking. Therefore, we also aimed to provide normative data for this scale. Data were collected among a representative sample of 326 participants (*M* age = 42.90 years, *SD* = 14.76; range 18–70) in Norway, of which 49.7% was female. Results yielded evidence for a six-factor structure (i.e., combining satisfaction/frustration with the type of need) and showed the subscales to be highly reliable. Subsequent structural equation modeling showed that both need satisfaction and need frustration related strongly to vitality, life satisfaction, and internalizing symptoms, but in opposite ways. Norm scores were provided, thereby differentiating between women and men and different age groups. These findings support the use of the Norwegian BPNSFS and provide researchers and professionals with normative data on the most widely used tool to assess individuals’ satisfaction and frustration of the basic psychological needs for autonomy, competence, and relatedness.

## Introduction

Self-determination theory (SDT) is a prominent theory within the field of motivation and human flourishing, thereby examining factors that contribute to individuals’ optimal functioning, performance, and well-being (Deci and Ryan, 2000; Ryan and Deci, 2017). Specifically, within the Basic Psychological Needs Theory (BPNT), one of SDT’s six mini-theories, a set of three innate and therefore universal psychological needs is said to be essential in understanding individuals’ psychological functioning. Although a vast number of studies, mostly employing rather homogeneous student samples, have indeed shown the satisfaction of these needs to be a key indicator of human well-being (Martela and Ryan, 2021), less is known about the role of these needs in a Norwegian context. This study, therefore, aimed to validate the Norwegian version of the Basic Psychological Need Satisfaction and Frustration Scale (BPNSFS; Chen et al., 2015) in a heterogenous sample. By employing a stratified sampling procedure, this study also aimed to contribute to extant research by providing norm scores with respect to the BPNSFS.

### Psychological need satisfaction and frustration

Within SDT, the three basic psychological needs of autonomy, competence, and relatedness are stated to be essential for individuals’ well-being and thriving (Deci and Ryan, 2000; Ryan and Deci, 2017). Autonomy encompasses feelings of volition and choice and is satisfied, for instance, when individuals feel that they can be themselves. Competence refers to a sense of success in daily activities, with competence satisfaction being apparent when individuals feel capable in reaching important goals. Finally, relatedness denotes experiencing closeness with other important individuals, with this need being satisfied when individuals care for and feel cared for by others. Need frustration, on the other hand, refers to feeling pressured to think, behave, or feel in a certain way (i.e., autonomy frustration), feeling like a failure (i.e., competence frustration), and experiencing social isolation or rejection (i.e., relatedness frustration; Deci and Ryan, 2000; Ryan and Deci, 2017).

Initial research focused on the benefits of need satisfaction for individuals’ thriving and demonstrated that need satisfaction was associated with a variety of indicators of well-being (see Vansteenkiste and Ryan, 2013 for an overview). More recently, there has been an increasing interest in the dysfunctional side of human development, with a focus on the concept of need frustration referring to the active obstruction and undermining of the psychological needs (Vansteenkiste and Ryan, 2013). The distinction between need satisfaction and need frustration is both conceptually and empirically justified, with these constructs not only being negatively related to one another but also displaying different antecedents and outcomes (Ryan et al., 2016). That is, in line with a hypothesized bright and dark path of human functioning (Vansteenkiste and Ryan, 2013), need satisfaction is most strongly related to adaptive outcomes (e.g., life satisfaction), whereas need frustration is more predictive of ill-being and psychopathology (e.g., depressive symptoms; Bartholomew et al., 2011; Ryan et al., 2016).

### The Basic Psychological Need Satisfaction and Frustration Scale

The BPNSFS (Chen et al., 2015) is the most widely used scale to determine individuals’ psychological need satisfaction and frustration and is currently available in 14 languages (Van der Kaap-Deeder et al., 2020), although a Norwegian version is currently lacking. The scale consists of 24 items reflecting satisfaction (12 items) and frustration (12 items) of each of the three psychological needs (8 items per need). Originally, the BPNSFS was validated in four different cultures (i.e., Belgium, Peru, China, and the United States; Chen et al., 2015), with later studies replicating these findings in several more countries (e.g., Germany, Portugal, Serbia, Italy; see also Van der Kaap-Deeder et al., 2020). Overall, these studies indicated evidence for a six-factor solution of the BPNSFS (e.g., Chen et al., 2015; Heissel et al., 2018), with each factor referring to the satisfaction or frustration of one of the three needs. Some studies also found support for a higher-order model with need satisfaction and need frustration as second-order factors and the satisfaction and frustration of each specific need as first-order factors (Chen et al., 2015; Frielink et al., 2019).

The importance of need-based experiences as assessed with the BPNSFS in individuals’ well-being and ill-being has emerged across cultures (Chen et al., 2015), in diverse life domains such as in the contexts of education, work, and sport (Ryan and Deci, 2017), and even for individuals attaching low importance to the satisfaction of these needs (Chen et al., 2015). Nonetheless, most of the extant research including the BPNSFS has employed rather homogenous samples such as student samples (e.g., Chen et al., 2015; Costa et al., 2015). This limits the generalizability of such previous findings, as students have been shown to differ significantly from a more general sample in terms of for instance educational level and age (e.g., Hanel and Vione, 2016). Thus, more research is needed including representative samples differing for instance in age, education, income, or employment status. There is some indirect evidence available comparing across studies indicating that, for instance, need satisfaction and frustration are crucial predictors of psychological functioning among both young and old adults (Mackenzie et al., 2017). Also, employing a large sample of Spanish adolescents, Rodríguez-Meirinhos et al. (2020) showed that independent of individuals’ gender, age, and SES, need satisfaction related to well-being, whereas need frustration was associated with psychological adjustment problems. Heissel et al. (2018) also demonstrated that need frustration was related and need satisfaction was unrelated to depressive symptoms across subsamples of university students, working adults, and senior adults, whereas the significance of the relation between need satisfaction and well-being depended on the type of subsample. Nonetheless, no study thus far employed a representative sample to examine the effects of need-based experiences in individuals’ well-being and ill-being.

The inclusion of a representative sample creates the opportunity to provide norm scores concerning the BPNSFS, which are currently lacking. Simply categorizing an individual’s score on the BPNSFS as high or low based on the possible scores (i.e., 1–5) is not meaningful. Indeed, on average, previous studies have shown quite high scores on need satisfaction (around 4) and relatively low scores on need frustration (around 2; e.g., Chen et al., 2015), emphasizing the need for norm scores to provide a more detailed account of individuals’ need-based experiences. Further, norm scores are not only essential when interpreting individuals’ unique level of need satisfaction and need frustration (e.g., in the clinical context), but can also be employed to compare need-based experiences across diverse cultures.

### The present study

Research examining the three psychological needs of autonomy, competence, and relatedness is increasing rapidly, thereby mostly employing the BPNSFS to determine individuals’ need satisfaction and frustration. This research aims to add to this extant research in three important ways. First, we aimed to validate the Norwegian version of the BPNSFS, thereby examining its dimensionality, internal consistency, and measurement invariance across gender and SES. Second, we investigated the predictive validity of the Norwegian BPNSFS, where it was expected that need satisfaction would relate especially to vitality and life satisfaction (positively) and to a lesser degree to internalizing symptoms (negatively), whereas an opposite pattern of relations was expected for need frustration. Third, by employing a representative sample (in terms of age, gender, and geographical location), we provided for the first time normative data on this scale. Such norm scores inform not only future research, but also facilitate the use of the BPNSFS by professionals (e.g., within a clinical context).

## Materials and methods

### Participants and procedure

Participants were recruited through a professional data collection organization (Norstat) that has access to a panel of 87,000 individuals within Norway. Membership in this panel is by invitation only to ensure random selection and representativeness of the members. In total, 3,585 members were invited through e-mail or push notification in the panel app to participate in this study, thereby also receiving information about the length of the survey (*ca.* 20 min) and the incentive. At the start of the survey, participants were informed that the data would be processed in a confidential way, that their participation was voluntary, and that they were entitled to terminate their participation at any moment. All participants completed an informed consent. To ensure that the sample would be representative in terms of age, gender, and geographical location within Norway, we employed a stratified sampling procedure. As an inclusion criterion, we only included individuals between the ages of 18 and 70 (as older individuals might experience difficulties in filling out the questionnaire online). Participants received 20 NOK (*ca.* 2 EUR) as a compensation for their participation, which they could redeem through, for instance, a variety of gift vouchers, lottery tickets, renting a movie online, or donating to a number of charity organizations.

In total, 326 individuals (*M* age = 42.90 years, *SD* = 14.76; range 18–70) participated in this study, of which 49.7% were female. With respect to the geographical location within Norway, 31.0% came from southeast Norway (i.e., Østlandet), 25.5% came from westernmost part of Norway (i.e., Vestlandet), 12.3% came from Oslo, 11.3% came from southern Norway (i.e., Sørlandet, Telemark, and Vestfold), 10.7% came from Mid-Norway (i.e., Midt-Norge), and 9.2% came from northern Norway (i.e., Nord-Norge). With regard to the highest completed educational level, 26.7% completed university/college up to 3 years (bachelor’s degree), 24.8% completed university/college 4 years or more (master’s degree and higher), 24.5% completed high school, 17.2% completed vocational school or another 1–2 year education after upper secondary school, 4.9% completed primary school, and 1.8% indicated to have completed another type of education. Most participants indicated that they were married or cohabitant (56.7%) or single (25.2%), with others indicating to have a boyfriend/girlfriend (9.8%), to be separated/divorced (6.4%), to be a widow(er) (0.6%), or to not want to disclose this information (1.2%). Finally, with respect to personal income, most individuals (45.7%) earned between 300,001 and 600,000 NOK (*ca.* 30,000 and 60,000 EUR).

### Measures

#### Socioeconomic status

Participants’ SES was determined by their educational level and income. After creating standardized scores for both variables, these were averaged together and recategorized thereby aiming to have three groups of approximately equal size. Specifically, group 1 (low SES; *n* = 110) scored below −0.40, group 2 (middle SES; *n* = 98) had scores from −0.40 to 0.37, and group 3 (high SES; *n* = 116) scored 0.37 or up.

#### Psychological need satisfaction and need frustration

To measure the satisfaction and frustration of the psychological needs, the Basic Psychological Need Satisfaction and Frustration Scale (BPNSFS; Chen et al., 2015) was used. The BPNSFS consists of 24 items, with 12 items reflecting need satisfaction (four items per need) and 12 items assessing need frustration (four items per need). Example items are “I feel a sense of choice and freedom in the things I undertake” (autonomy satisfaction), “I am confident that I can do things well” (competence satisfaction), and “I feel connected to people who care about me and who I also care about” (relatedness satisfaction). Items were rated on a 5-point Likert scale, ranging from 1 (*Completely disagree*) to 5 (*Completely agree*). Items were translated from English to Norwegian by two researchers and, subsequently, translated back to English by a third researcher. All three researchers were highly familiar with SDT and the three psychological needs of autonomy, competence, and relatedness in particular. Discrepancies between the translations were discussed by these three researchers as to come to the final wording of the items.

#### Vitality

Participants’ degree of perceived vitality was assessed with the Subjective Vitality Scale (Ryan and Frederick, 1997). We selected three positively worded and face valid items of this 7-item scale, namely: “I feel alive and vital,” “I nearly always feel alert and awake,” and “I feel energized.” Items were rated on a 7-point Likert scale, ranging from 1 (*Not true at all*) to 7 (*Very true*). This scale had an adequate reliability (*α* = 0.89).

#### Life satisfaction

To assess participants’ life satisfaction, we employed the Satisfaction with Life Scale (Diener et al., 1985). Again, the three most face valid items were selected (out of the five original items): “In most ways my life is close to my ideal,” “The conditions of my life are excellent,” and “I am satisfied with my life.” Items were rated on a 7-point Likert scale, ranging from 1 (*Does not fit at all*) to 7 (*Fits perfectly*). This scale showed an excellent reliability (*α* = 0.91).

#### Internalizing symptoms

The Hopkins Symptom Checklist-25 (HSCL-25; Derogatis et al., 1974) was employed to determine participants’ level of depressive (15 items) and anxiety (10 items) symptoms. Example items are “Feelings of worthlessness” (depressive symptoms subscale) and “Being scared for no reason” (anxiety symptoms subscale). Participants were asked to indicate how much each of these symptoms was bothering them in the last 14 days. Their responses were scored on a scale from 1 (*Not at all*) to 4 (*A lot*). Given that the two sets of items were highly correlated (*r* = 0.78), they were averaged to form a composite score, which we refer to as “internalizing symptoms” (*α* = 0.95).

### Plan of analyses

#### Internal structure

After examining the descriptives of and correlations between the study variables, we investigated the internal structure of the Norwegian version of the BPNSFS by estimating models using MPlus 8.4 (Muthén and Muthén, 1998–2017) through a robust maximum-likelihood approach. First, *via* confirmatory factor analysis (CFA), we compared four models: (1) a 6-factor model differentiating between the satisfaction and frustration of each need; (2) a 3-factor model representing the needs for autonomy, competence and relatedness; (3) a 2-factor hierarchical model (6^2^-factor) including six first-order (same as the first model) and two second-order factors (need satisfaction and need frustration) (4) and a 3-factor hierarchical model (6^3^-factor) including six first-order factors (same as the first model) and three second-order factors (autonomy, competence and relatedness). These CFA models were compared by means of the Satorra-Bentler Scaled chi-square difference test (SBS-*χ*^2^; Satorra and Bentler, 1994), while also considering the Akaike’s Information Criterion (AIC) and the Bayesian Information Criterion (BIC) (smaller values preferable; Kline, 2015). Second, reliabilities of all subscales were calculated.

#### Measurement invariance

Third, we examined the measurement invariance of the best fitting CFA model across participants’ gender (female, male) and SES (low, middle, high) in a series of multigroup CFAs with three levels of invariance: configural (i.e., no equality constraints), weak (i.e., equal factor loadings across groups) and strong (i.e., equal item intercepts in addition to previous constraints) invariance (Chen, 2007).^1^ Models were compared using the ΔSBS*χ*2 for two nested models, changes in fit indices, and AIC and BIC values. For testing weak invariance, a change of ≥−0.01 in CFI, supplemented by a change of ≥0.03 in SRMR or a change of ≥0.015 in RMSEA, would indicate non-invariance. When testing strong invariance, a change of ≥−0.01 in CFI, supplemented by a change of ≥0.015 in SRMR or a change of ≥0.015 in RMSEA, would indicate non-invariance. Among the three indices, CFI is chosen as the main criterion (Chen, 2007).

#### Predictive validity and normative analyzes

Fourth, after examining the effects of the background variables, we ran a model with need satisfaction and need frustration as predictors of vitality, life satisfaction, and internalizing symptoms. To evaluate model fit, we employed several indices: the *χ*^2^ test, the comparative fit index (CFI), the standardized root mean square residual (SRMR), and the root mean square error of approximation (RMSEA). An acceptable to good fit was indicated by *χ*^2^/*df* ratio of 3 (acceptable)/2 (good) or below, CFI values of 0.90 (acceptable)/0.95 (good) or above, SRMR values of 0.10 (acceptable)/0.05 (good) or below, and RMSEA values of 0.10 (acceptable)/0.05 (good) or below (Browne and Cudeck, 1992; Hu and Bentler, 1999; Kline, 2015). Finally, after examining the relations between the background characteristics and the satisfaction and frustration of each of the needs, we calculated norm scores. There were no missing data.

## Results

### Preliminary analyzes

Descriptives of and correlations between the study variables can be found in Table 1. First, satisfaction of all three needs related positively to vitality and life satisfaction and negatively to internalizing symptoms, whereas need frustration showed an opposite pattern of relations. Autonomy, relatedness, and competence satisfaction were mutually positively correlated, as were the three subscales of need frustration. Additionally, satisfaction of each need was negatively correlated with frustration of the same need.

**Table 1:** Descriptives of and correlations between the study variables (*N* = 326).

	*M*	*SD*	1	2	3	4	5	6	7	8	9	10
1. Need satisfaction	3.77	0.63	-									
2. Autonomy satisfaction	3.53	0.75	0.87	-								
3. Competence satisfaction	3.79	0.78	0.85	0.68	-							
4. Relatedness satisfaction	4	0.76	0.77	0.49	0.44	-						
5. Need frustration	2.41	0.79	−0.74	−0.67	−0.65	−0.53	-					
6. Autonomy frustration	2.8	0.94	−0.54	−0.58	−0.45	−0.33	0.85	-				
7. Competence frustration	2.41	0.99	−0.72	−0.64	−0.75	−0.4	0.87	0.61	-			
8. Relatedness frustration	2.01	0.86	−0.62	−0.46	−0.43	−0.65	0.82	0.55	0.58	-		
9. Vitality	4.08	1.38	0.62	0.57	0.54	0.44	−0.6	−0.56	−0.54	−0.43	-	
10. Life satisfaction	4.19	1.43	0.67	0.6	0.53	0.54	−0.58	−0.44	−0.52	−0.51	0.66	-
11. Internalizing symptoms	1.79	0.62	−0.55	−0.51	−0.52	−0.34	0.67	0.61	0.6	0.5	−0.62	−0.55

All correlations were significant at the *p* < 0.001 level.

### Primary analyzes

#### Confirmatory factor analyzes

CFAs with robust maximum likelihood estimation were performed to evaluate the fit of the 6-factor, 3-factor, 6^2^-factor, and 6^3^-factor models. Despite increasing the number of iterations, the 6^3^-factor model did not converge. The fit indices of the other models are displayed in Table 2. The 6-factor model showed a good and significantly better fit than the 6^2^-factor (acceptable fit) and the 3-factor (poor fit) model. Parameter estimates of the CFA for the 6-factor model are presented in Table 3. All standardized factor loadings were above 0.63 and significant at the *p* < 0.001 level, and the robust standard errors were small (ranging from 0.03 to 0.05). With respect to the correlations among the six latent variables, results showed that the satisfaction of each need was strongly and negatively related to the frustration of this need (autonomy: −0.66; competence: −0.88; relatedness: −0.77). Moreover, all three indicators of need satisfaction were positively related to one another (range = [0.51, 0.82]) as were the three indicators of need frustration (range = [0.64, 0.71]). The correlations between the satisfaction of one specific need and the frustration of an other need were all negative (range = [−0.46, −0.76]).

**Table 2:** Goodness-of-fit indices of the tested models.

	*χ* ^2^	*df*	*χ*^2^/*df*	CFI	SRMR	RMSEA	AIC	BIC	Model comparison
						(90% CI)			ΔSBS-*χ*^2^ (Δ*df*)
6-Factor model	409.727^**^	237	1.73	0.948	0.05	0.047 (0.039, 0.055)	18,361	18690.4	
3-Factor model	742.281^***^	249	2.91	0.851	0.074	0.078 (0.072, 0.084)	18766.2	19050.2	357.70 (12)^***^
6^2^-Factor hierarchical model	545.609^***^	245	2.23	0.909	0.077	0.061 (0.054, 0.068)	18520.4	18819.6	143.26 (8)^***^

CFI, Comparative Fit Index; SRMR, Standardized Root Mean Square Residual; RMSEA, Root Mean Square Error of Approximation; AIC, Akaike’s Information Criterion; BIC, Bayesian Information Criterion; ΔSBS*χ*^2^ = Satorra-Bentler scaled Chi-square difference. Models were compared with the 6-factor model.

** *p* < 0.01;

*** *p* < 0.001.

**Table 3:** Factor loadings, communalities, items means, and standard deviations of the 6-factor CFA.

Items	Satisfaction			Frustration			*R^2^*	*M*	*SD*
	Aut	Comp	Relate	Aut	Comp	Relate			
I feel a sense of choice and freedom in the things I undertake	0.63						0.4	3.74	0.93
I feel that my decisions reflect what I really want	0.77						0.6	3.48	0.92
I feel my choices express who I really am	0.71						0.51	3.51	0.9
I feel I have been doing what really interests me	0.72						0.52	3.37	1.04
I feel confident that I can do things well		0.77					0.6	3.92	0.92
I feel capable at what I do		0.8					0.64	3.85	0.92
I feel competent to achieve my goals		0.79					0.62	3.71	1.01
I feel I can successfully complete difficult tasks		0.73					0.53	3.67	0.89
I feel that the people I care about also care about me			0.79				0.62	4.04	0.94
I feel connected with people who care for me, and for whom I care			0.73				0.54	4.12	0.9
I feel close and connected with other people who are important to me			0.81				0.66	3.99	0.94
I experience a warm feeling with the people I spend time with			0.71				0.51	3.87	0.9
Most of the things I do feel like “I have to”				0.77			0.59	3.16	1.03
I feel forced to do many things I would not choose to do				0.77			0.59	2.61	1.1
I feel pressured to do too many things				0.76			0.51	2.6	1.14
My daily activities feel like a chain of obligations				0.82			0.68	2.83	1.21
I have serious doubts about whether I can do things well					0.76		0.58	2.17	1.09
I feel disappointed with many of my performance					0.78		0.61	2.55	1.11
I feel insecure about my abilities					0.84		0.71	2.57	1.18
I feel like a failure because of the mistakes I make					0.78		0.61	2.37	1.27
I feel excluded from the group I want to belong to						0.73	0.53	2.03	1.1
I feel that people who are important to me are cold and distant toward me						0.79	0.62	1.88	1
I have the impression that people I spend time with dislike me						0.74	0.55	1.83	0.98
I feel the relationships I have are just superficial						0.74	0.55	2.28	1.12

Aut, Autonomy; Comp, Competence; Relate, Relatedness.

#### Reliabilities

Although Cronbach’s alpha is the most common mean of assessing internal consistency, it has also received important criticism (Dunn et al., 2014). Therefore, we calculated both Cronbach’s alpha’s (for reasons of comparison with previous research) and McDonald’s omega (*ω*) (Raykov, 1997) to evaluate the reliability of the BPNSFS subscales. Results showed that both need satisfaction (*α* = 0.89; 95% *CI* [0.87, 0.91]; *ω* = 0.89; 95% *CI* [0.86, 0.91]) and frustration (*α* = 0.91; 95% *CI* [0.89, 0.92]; *ω* = 0.91; 95% *CI* [0.89, 0.92]) displayed an adequate internal consistency. Reliabilities for the need-specific subscales were also satisfactory: *α* = 0.80; 95% *CI* [0.76, 0.83]/*ω* = 0.80; 95% *CI* [0.75, 0.84] (autonomy satisfaction), *α* = 0.85; 95% *CI* [0.83, 0.88]/*ω* = 0.86; 95% *CI* [0.82, 0.88] (competence satisfaction), *α* = 0.85; 95% *CI* [0.82, 0.87]/*ω* = 0.85; 95% *CI* [0.81, 0.88] (relatedness satisfaction), *α* = 0.86; 95% *CI* [0.84, 0.88]/*ω* = 0.86; 95% *CI* [0.83, 0.89] (autonomy frustration), *α* = 0.87; 95% *CI* [0.84, 0.89]/*ω* = 0.87; 95% *CI* [0.84, 0.90] (competence frustration), and *α* = 0.83; 95% *CI* [0.80, 0.86]/*ω* = 0.83; 95% *CI* [0.79, 0.86] (relatedness frustration).

#### Measurement invariance

In a next step, we examined the measurement invariance of the BPNSFS 6-factor solution across participants’ gender (Table 4) and SES (Table 5). To test the measurement invariance of the model across gender, measurement invariance of the 6-factor model was tested in a series of multigroup CFAs with three levels of invariance (configural, weak and strong invariance). When calculating the 6-factor model for the two subsamples separately, fit indices remained acceptable. Further, overall weak measurement invariance could be stablished with the constrained model not differing significantly from the unconstrained model, and the CFI, SRMR and RMSEA indices for the constrained and unconstrained models being not substantially different. As for strong measurement invariance, the constrained model differed significantly from the unconstrained model, but the difference in the fit indices (CFI, SRMR and RMSEA) for the constrained and unconstrained models were not substantially different.

**Table 4:** Confirmatory factor analyzes for gender and invariance testing.

	*χ* ^2^	*df*	*χ*^2^/*df*	*χ*^2^ (Δ*df*)	CFI	ΔCFI	SRMR	ΔSRMR	RMSEA	ΔRMSEA	Model comparison
											ΔSBS-*χ*^2^ (Δ*df*)
*Gender*											
Female (*n* = 162)	404.64^***^	237	1.71^g^		0.923^a^		0.061^a^		0.071^a^		
Male (*n* = 164)	331.28^***^	237	1.40^g^		0.941^a^		0.059^a^		0.055^a^		
*Invariance level*											
Configural	733.63^***^	474	1.55		0.931		0.06		0.063		
Weak	751.61^***^	492	1.53	17.98 (18)	0.931	0.000	0.066	0.006	0.062	−0.001	16.92 (18)
Strong	790.46^***^	510	1.55	38.85 (18)	0.926	−0.005	0.068	0.002	0.063	0.001	41.22 (18)^**^

CFI, Comparative Fit Index; SRMR, Standardized Root Mean Square Residual; RMSEA, Root Mean Square Error of Approximation; ΔCFI, ΔSRMR, and ΔRMSEA refer to changes in, respectively, CFI, SRMR, and RMSEA when compared to the baseline model; ΔSBS*χ*^2^ = Satorra-Bentler scaled Chi-square difference; n refers to the sample size.

a Acceptable value.

g Good value.

n Unacceptable value.

**p < 0.001;

*** *p* < 0.001.

**Table 5:** Confirmatory factor analyzes for socioeconomic status (SES) subsamples and invariance testing.

	*χ* ^2^	*df*	*χ*^2^/*df*	*χ*^2^ (Δ*df*)	CFI	ΔCFI	SRMR	ΔSRMR	RMSEA	ΔRMSEA	Model comparison
											ΔSBS-*χ*^2^ (Δ*df*)
*SES sample*											
Low (*n* = 110)	365.83^***^	237	1.54^g^		0.890^n^		0.075^a^		0.077^a^		
Middle (*n* = 98)	310.24^***^	237	1.31^g^		0.939^a^		0.065^a^		0.058^a^		
High (*n* = 116)	367.47^***^	237	1.55^g^		0.920^a^		0.070^a^		0.071^a^		
*Invariance level*											
Configural	1046.204^***^	711	1.47		0.915		0.07		0.069		
Weak	1077.400^***^	747	1.44	31.20 (36)	0.917	0.002	0.074	0.004	0.067	−0.002	29.75 (36)
Strong	1136.564^***^	783	1.45	59.16 (36)	0.912	−0.005	0.076	0.002	0.076	0.009	59.85 (36)^**^

CFI, Comparative Fit Index; SRMR, Standardized Root Mean Square Residual; RMSEA, Root Mean Square Error of Approximation; ΔCFI, ΔSRMR, and ΔRMSEA refer to changes in, respectively, CFI, SRMR, and RMSEA when compared to the baseline model; ΔSBS*χ*^2^, Satorra-Bentler scaled Chi-square difference.

a Acceptable value.

g Good value.

n Unacceptable value.

** *p* < 0.01;

*** *p* < 0.001.

With respect to SES, when calculating the 6-factor model for the three subsamples separately, fit indices remained acceptable. Further, weak measurement invariance could be established with the constrained model not differing significantly from the unconstrained model, and the CFI, SRMR and RMSEA being comparable across both models. As for strong measurement invariance, the constrained model differed significantly from the unconstrained model, although the values of the fit indices CFI, SRMR and RMSEA did not differ substantially for the two models.

#### Predictive validity

Before investigating need-based experiences as predictors of participants’ well-being and ill-being, we first examined the relation between the background characteristics and the outcomes. A MANCOVA was performed with gender, marital status, and location as fixed factors, age, education and income as covariates, and the indicators of well-being and ill-being as outcomes. Results showed that age (*F*(3, 258) = 5.03, *p* = 0.002, *η*^2^ = 0.06), gender (*F*(3, 258) = 6.17, *p* < 0.001, *η*^2^ = 0.07), income (*F*(3, 258) = 4.90, *p* = 0.003, *η*^2^ = 0.05), and marital status (*F*(9, 628) = 4.82, *p* < 0.001, *η*^2^ = 0.05) significantly related to the outcomes, although displaying only small to medium effect sizes (Cohen, 1988; Cohen et al., 2003). Specifically, we found that older participants and those with a higher income reported higher levels of vitality and life satisfaction and a lower level of internalizing symptoms. Additionally, women reported a higher level of internalizing symptoms than men. Married or cohabiting participants experienced more life satisfaction than those who reported being single or having a boyfriend/girlfriend. Based on these findings, we controlled for age, gender, marital status, and income in the subsequent analyzes.

**Figure 1:**
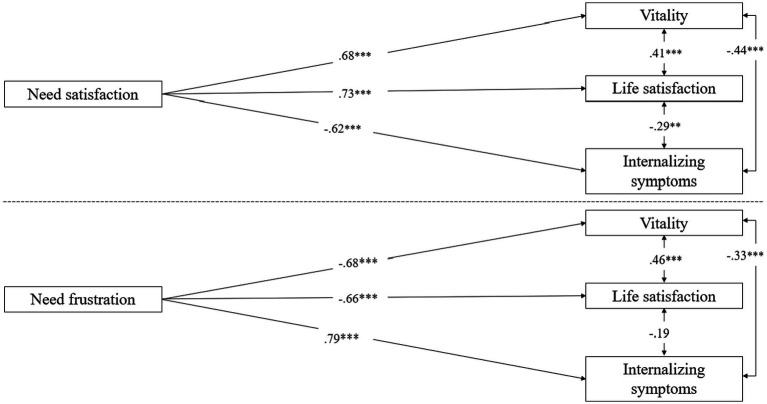
Structural model depicting the relation between need-based experiences and indicators of well-being and Ill-being. The measurement model and the effects of the control variables are not shown for reasons of clarity. The dashed line separates the two tested models with need satisfaction or need frustration as predictor of the outcomes. ^**^p < 0.01; ^***^p < 0.001.

Subsequently, we examined the value of need satisfaction and frustration in predicting indicators of well-being and ill-being. To avoid multicollinearity (due to the strong negative correlation between need satisfaction and need frustration), we modeled two separate structural models with pathways from need satisfaction (Model 1) and with pathways from need frustration (Model 2) to the outcomes. With respect to the outcomes, we modeled vitality, life satisfaction, and internalizing symptoms as three separate latent factors. In doing so, the two well-being factors were indicated by their respective items. With respect to the internalizing symptoms, we employed the item-to-construct balance method (Landis et al., 2000), where stronger loading items are combined with weaker loading items, resulting in six parcels (each representing the average of 4 or 5 items). Both the model involving need satisfaction (*χ*^2^/*df* = 2.01; CFI = 0.92; SRMR = 0.10; RMSEA = 0.06) and the model involving need frustration (*χ*^2^/*df* = 1.93; CFI = 0.94; SRMR = 0.09; RMSEA = 0.06) fitted the data adequately. As displayed in Figure 1, need satisfaction related strongly and positively to vitality and life satisfaction and negatively to internalizing symptoms, with need frustration showing an opposite pattern of relations.

#### Normative analyzes

First, a MANCOVA was performed with gender, marital status, and location as fixed factors, age, education and income as covariates, and autonomy, competence, and relatedness satisfaction and frustration as the six outcomes. Results showed that age (*F*(6, 255) = 3.04, *p* = 0.007, *η*^2^ = 0.07), gender (*F*(6, 255) = 7.37, *p* < 0.001, *η*^2^ = 0.15), and income (*F*(6, 255) = 4.79, *p* < 0.001, *η*^2^ = 0.10) significantly related to the outcomes (and showing medium to large effect sizes), whereas the effects of marital status, location, and education were non-significant. Specifically, older (compared to younger) individuals indicated a higher level of competence satisfaction and lower levels of autonomy, competence, and relatedness frustration. Further, women experienced significantly more relatedness satisfaction and less relatedness frustration than men. Finally, a higher income related to more competence satisfaction and less competence and relatedness frustration. Since there were significant differences based on age and gender in the satisfaction and frustration of the three needs, norm scores were provided separately for women and men per age group (Table 6). No further differentiation based on income was made, to ensure that the number of participants within each cell was not too small.

**Table 6:** Norm scores and sample sizes for need satisfaction and frustration for women and men by age.

Age (years)	18–29		30–44		45–59		60–70	
	W *M* (*SD*) (*N* = 47)	M *M* (*SD*) (*N* = 34)	W *M* (*SD*) (*N* = 45)	M *M* (*SD*) (*N* = 45)	W *M* (*SD*) (*N* = 45)	M *M* (*SD*) (*N* = 57)	W *M* (*SD*) (*N* = 25)	M *M* (*SD*) (*N* = 28)
Need satisfaction	3.61 (0.73)	3.41 (0.56)	3.68 (0.58)	3.64 (0.61)	4.04 (0.60)	3.84 (0.56)	4.03 (0.64)	4.05 (0.52)
Autonomy	3.41 (0.74)	3.27 (0.68)	3.33 (0.76)	3.39 (0.73)	3.70 (0.83)	3.60 (0.67)	3.84 (0.79)	3.86 (0.63)
Competence	3.44 (0.90)	3.51 (0.67)	3.57 (0.83)	3.71 (0.75)	4.01 (0.84)	4.10 (0.55)	3.95 (0.71)	4.07 (0.58)
Relatedness	4.00 (0.80)	3.44 (0.74)	4.14 (0.73)	3.81 (0.68)	4.42 (0.51)	3.82 (0.79)	4.30 (0.73)	4.21 (0.64)
Need frustration	2.74 (0.81)	2.70 (0.70)	2.65 (0.73)	2.59 (0.74)	2.10 (0.76)	2.26 (0.75)	2.07 (0.75)	1.91 (0.60)
Autonomy	3.07 (0.90)	2.89 (0.82)	3.23 (0.88)	2.92 (0.87)	2.61 (0.93)	2.70 (0.97)	2.42 (1.00)	2.18 (0.81)
Competence	2.87 (1.07)	2.83 (0.87)	2.62 (0.96)	2.71 (0.97)	2.08 (1.03)	2.06 (0.80)	2.14 (0.92)	1.84 (0.55)
Relatedness	2.27 (0.81)	2.38 (0.93)	2.11 (0.91)	2.12 (0.83)	1.61 (0.67)	2.02 (0.91)	1.64 (0.62)	1.71 (0.76)

W, Women; M, Men. We also examined measurement invariance with regard to age, after creating four categories (i.e., 18–29, 30–44, 45–59, and 60–70). Unfortunately, we did not obtain sufficient fit for these models, probably due to having too few participants per group.

## Discussion

An increasing amount of research has now indicated that the basic psychological needs are crucial in understanding both individuals’ well-being and striving as well as their ill-being and even psychopathology (Vansteenkiste and Ryan, 2013). Research on the effects of need-based experiences in representative, heterogeneous samples is, however, scarce and there is a need for norm scores to increase the applicability of the BPNSFS in professional (e.g., clinical) contexts. Given that no previous study formally validated a Norwegian version of the BPNSFS, we also aimed to create and establish the dimensionality and internal consistency of the Norwegian BPNSFS.

Results concerning the validation of the Norwegian BPNSFS showed this scale to be reliable, with the reliabilities of the need satisfaction and need frustration subscales being excellent as well as the reliabilities related to the six specific subscales (i.e., type of need X satisfaction/frustration). Further, CFA analyzes indicated that the best-fitting model was the 6-factor model and, to a somewhat lesser degree, the 6^2^-model, which both distinguish between the satisfaction and frustration dimensions of the three needs. These two models showed a superior fit compared to the 3-factor model, which does not explicitly include the distinction between satisfaction and frustration of the needs. These findings are in line with theoretical assumptions (Vansteenkiste et al., 2020) and with the results found in previous validation studies in other languages (Chen et al., 2015; Heissel et al., 2018). The fit of the 6^2^-factor model was worse compared to the 6-factor model. This result was expected according to the methodological and theoretical assumptions, and possibly derives from the need-specific variance shared by items of different latent factors. Heissel et al. (2018) also found a worse fit for the 6^2^-factor hierarchical model compared to the 6-factor model for the 24-item version of the German BPNSFS. Similar results were found in previous international studies that showed a worse fit for the hierarchical models compared to the reduced 6-factor model (e.g., Chen et al., 2015; Liga et al., 2020). Still, the 6^2^-factor hierarchical model is feasible, as the model showed an acceptable fit, and supports the distinction between need satisfaction and need frustration as different dimensions (Frielink et al., 2019).

Similar to previous research examining measurement invariance of the BPNSFS across different adult age groups (Heissel et al., 2018) and across age, gender, and SES among adolescents (Rodríguez-Meirinhos et al., 2020), we examined invariance related to gender and SES in a heterogeneous adult population (note that age was not reported here, due to the sample size being too small). In line with SDT’s universality claim, we found the six-factor model to be invariant across gender and SES, with the fit indices being comparable for the constrained and unconstrained models.

A further aim of this study was to examine the predictive value of need satisfaction and need frustration, thereby focusing on indicators of well-being and ill-being as outcomes. Both need satisfaction and need frustration were found to be strongly related to the outcomes. Specifically, whereas need satisfaction related positively to vitality and life satisfaction and negatively to internalizing problems, need frustration displayed an opposite pattern of relations. Thus, herein we did not find evidence for the bright and dark path of need-based experiences (Vansteenkiste and Ryan, 2013), as both need satisfaction and need frustration were strongly related to both well-being and ill-being. Unfortunately, due to the high correlation between need satisfaction and need frustration, we were not able to examine the unique predictive value of need satisfaction and need frustration in one model. Moreover, these results should be interpreted with caution given that both our predictors (i.e., need satisfaction and need frustration) and our outcomes were assessed at the same time.

We also found some interesting differences in mean-levels of need satisfaction and frustration depending on individuals’ age, gender, and SES. Although previous research indicated that older individuals especially experience more autonomy (e.g., Stenling et al., 2021), we found that older individuals experienced less frustration regarding all three needs and also indicated a higher level of competence satisfaction than their younger counterparts. These findings fit with the Socioemotional Selectivity Theory (Carstensen et al., 2003), which states that with increasing age people become more skilled in enhancing their current well-being through for instance positively reappraising negative events (e.g., need-frustrating events). Results further showed that women and men only differed with respect to experienced relatedness, with women reporting more relatedness satisfaction and less relatedness frustration. Finally, those with a higher SES indicated to experience more competence satisfaction and less competence and relatedness frustration. Given these important differences, we presented norm scores separately for women and men while differentiating between different age groups. To avoid a too small number of individuals per cell, no further differentiation based on income was made.

### Limitations, directions for future research, and practical implications

This study had important strengths, such as the employment of a relatively large representative sample, reflecting variability in for instance age, gender, SES, and geographical location. This is also the first study to formally validate a Norwegian version of the BPNSFS and to provide norm scores. Nonetheless, the results of this study should be interpreted with caution, given several important limitations. First, this study made use of a cross-sectional design, which precludes causal inferences and does not provide information about changes in need-based experiences and outcomes across time. Further, this study did not include data from other important sources (e.g., well-being reported by close friends or family members) but was solely based on self-reports. To avoid common method biases (which increases the risk for socially desirable responding), an interesting avenue for future research is to use multiple measurement methods besides self-report, such as other-report and observation (Podsakoff et al., 2003). Moreover, future research could employ exploratory structural equation (ESEM) where the measurement model of the latent variables is evaluated through exploratory factor analysis, which has been found to better adjust for cross-factor loadings (Mai et al., 2018). Given that the BPNSFS employs a Likert scale, future research could explore, through sensitivity analyzes, other estimation methods that take into account categorical data such as the weighted least squares with mean and variance adjusted (WLSMV) (see also Liang and Yang, 2014). Finally, this study did not take into account whether participants were careless in their responding, a factor that needs to be considered in future research.

Given that this is the first study to examine norm scores of the BPNSFS among a non-clinical, representative sample, current findings provide valuable insights for clinical practice. That is, professionals could determine patients’ or clients’ need satisfaction and especially need frustration through the BPNSFS and compare these scores with the herein provided norm scores. This is especially important as the psychological needs are increasingly recognized to play a transdiagnostic in several forms of psychopathology (Vansteenkiste and Ryan, 2013).

## Conclusion

This study provided evidence for adequate psychometric properties of the Norwegian BPNSFS within a relatively large representative sample, reflecting variability in age, gender, SES, and geographical location and provided norm scores per gender and age group. Results especially pointed toward a six-factor solution differentiating between the three needs (autonomy, competence, relatedness) and two dimensions (satisfaction, frustration). In terms of its predictive validity, both need satisfaction and need frustration were found to be strongly related to indicators of well-being and ill-being in expected ways.

## Data availability statement

The raw data supporting the conclusions of this article will be made available by the authors, without undue reservation.

## Ethics statement

Ethical review and approval was not required for the study on human participants in accordance with the local legislation and institutional requirements. The patients/participants provided their written informed consent to participate in this study.

## Author contributions

JK-D took the lead in the study design and data collection. She also analyzed the data and wrote a first draft of the paper. AS conducted part of the analyzes. All authors, including MJ, FS, IS-L, and AH provided thorough feedback on the analyzes and writing of the text. All authors contributed to the article and approved the submitted version.

## Conflict of interest

The authors declare that the research was conducted in the absence of any commercial or financial relationships that could be construed as a potential conflict of interest.

## Publisher’s note

All claims expressed in this article are solely those of the authors and do not necessarily represent those of their affiliated organizations, or those of the publisher, the editors and the reviewers. Any product that may be evaluated in this article, or claim that may be made by its manufacturer, is not guaranteed or endorsed by the publisher.

## Footnotes

^1^We also examined measurement invariance with regard to age, after creating four categories (i.e., 18-29, 30-44, 45-59, and 60-70). Unfortunately, we did not obtain sufficient fit for these models, probably due to having too few participants per group.
